# An automated system of intrusion detection by IoT-aided MQTT using improved heuristic-aided autoencoder and LSTM-based Deep Belief Network

**DOI:** 10.1371/journal.pone.0291872

**Published:** 2023-10-04

**Authors:** P. M. Vijayan, S. Sundar

**Affiliations:** School of Electronics Engineering (SENSE), Vellore Institute of Technology, Vellore, Tamil Nādu, India; York St John University, UNITED KINGDOM

## Abstract

The IoT offered an enormous number of services with the help of multiple applications so it faces various security-related problems and also heavy malicious attacks. Initially, the IoT data are gathered from the standard dataset as Message Queuing Telemetry Transport (MQTT) set. Further, the collected data are undergone the pre-processing stage, which is accomplished by using data cleaning and data transformation. The resultant processed data is given into two models named (i) Autoencoder with Deep Belief Network (DBN), in which the optimal features are selected from Autoencoder with the aid of Modified Archimedes Optimization Algorithm (MAOA). Further, the optimal features are subjected to the AL-DBN model, where the first classified outcomes are obtained with the parameter optimization of MAOA. Similarly, (ii) Long Short-Term Memory (LSTM) with DBN, in this model, the optimal features are chosen from LSTM with the aid of MAOA. Consequently, the optimal features are subjected into the AL-DBN model, where the second classified outcomes are acquired. Finally, the average score is estimated by two outcomes to provide the final classified result. Thus, the findings reveal that the suggested system achieves outstanding results to detect the attack significantly.

## 1. Introduction

Intrusion detection in the network is considered an essential element in mobile security nowadays. Neural network applications have become very popular for intrusion detection networks [1]. However, due to minimal IoT devices, effective intrusion detection is essential along with an effective and lightweight framework [2]. Existing neural network frameworks for intrusion detection are not suitable for IoT devices because they utilized a large number of parameters in the system [3]. IoT is a network that connects actuators, sensors and also transfers the data through the internet by utilizing a processor [4]. Most IoT applications are connected to vehicles, smart cities, intelligent buildings, retail environments, and other systems that are vulnerable to attacks [5]. Therefore, it is highly important to enhance security like device authentication, secure booting, and access control. The continuous advancement of internet technology globally has led to a constant increase in attacks on networks [6]. Attackers exploit viruses, security defects, and vulnerabilities presented in the system network and, are used for attacking the system, accessing the website data, personal information of users, and so on [7].

Especially, various expansions occurred in industrial IoT devices are vulnerable to exposing privacy and also security threats, leading to a huge number of attackers spreading malicious data. This has motivated the researchers to analyze IoT security [8]. However, it is unavoidable for inducing network security issues in the smart grid due to different connections provided over power and communication resources [9]. Intrusion detection systems are considered mitigation and detection models and they involve random uncertainty-based communication noise [10]. General issues based on network security in the smart grid framework are summarized, and different classifications of smart grid attacks are offered [11]. The grid framework clearly displayed that massive network threats bring huge losses and damage to the quality of life and security of public property. Therefore, different analysis is highly important for intrusion detection systems in abnormal network attack [12].

Most IoT communication system is developed on the basis of Hypertext Transfer Protocol (HTTP). However, these networks often experience significant latency and utilize a large amount of network resources [12]. Similarly, the MQTT protocol utilizes minimal payload overhead along with multiple Quality of Service (QoS) and uses reduced network resources to overcome the issues presented in the HTTP protocol. IBM developed and published an open-source protocol named MQTT to transfer the message over client-server transport [13]. Most of the features of MQTT are utilized in limited communication environments for applications like Machine to Machine (M2M) and IoT and it needs a reduced code footprint [14]. The MQTT protocol is referred to as a binary and lightweight protocol because small overhead packets are available to transfer the data through the wire, and they are contrasted over conventional protocols such as HTTP [15]. The subscribe/publish pattern provides different ideas to an existing client-server framework, where the client communicates with the endpoint user directly. To optimize the security of IoT devices, researchers have proposed various approaches, including the use of deep learning methods. These methods are used for user behavior analysis, privacy preservation, vulnerability detection, and intrusion detection [16]. Deep learning approaches like Convolutional Neural Networks (CNNs) are utilized to extract, identify, detect, and learn complex features as well as patterns directly from raw IoT data. The utility rate of the device is enhanced for detecting the possible threats and attacks effectively in the IoT environment [17]. Deep learning models are highly efficient at the time of performing automatic feature extraction compared to conventional machine learning approaches which require more handcrafted statistical features. In past years, professionals developed various techniques for IDS systems but they face several complexities, leading to the design of a new framework IDS system to occur an effective intrusion detection rate in IoT devices.

The main contributions of the implemented IDS framework are described as follows.

- To build a novel intrusion detection model with the help of deep learning models as well as heuristic approaches to attain an efficient intrusion detection rate in IoT devices with the MQTT set for providing efficient data transmission with high security.
- To assure a new classification approach termed AL-DBN to detect intrusions presented in IoT devices with parameter optimization of LSTM, DBN, and autoencoder with the help of proposed MAOA. To maximize the accuracy rate of intrusion detection.
- To design a heuristic approach named MAOA to optimize the hidden neurons in LSTM and DBN and also epochs in autoencoder and DBN for maximizing the performance rate in intrusion detection effectively.
- To validate the effectiveness of the suggested intrusion detection model along with different conventional approaches and classifiers.

The developed approach for the intrusion detection model can be broken down into several phases. Phase 2 elaborates on different conventional related works associated with the IDS scheme. Phase 3 describes the characteristics of the MQTT set. Phase 4 explains the structure of the developed IDS model. Phase 5 represents the improved intrusion-detected model with several classifiers. In phase 6, various experimental analyses performed in the suggested model are explained and final phase 7 concludes the intrusion detection model.

## 2. Literature survey

### 2.1 Related works

In 2022, Siddharthan *et al*. [18] proposed an IDS system for predicting cyber-attacks with the help of Elite Machine Learning algorithms (EML). They also utilized a lightweight protocol to manage time-constrained problems. The developed model was validated using a test-bed setup along with hardware, here, the different sensor was connected by utilizing the MQTT protocol. The developed model was classified into three different classes such as sensor data collection, generation of multi-context feature with statistical cascade feature, and validation of dataset with different approaches. Multiple analyses were performed on the developed model and it achieved effective attack classification in terms of accuracy. In 2020, Eskandari et al. [19] developed a Passban-based IDS system to protect directly connected IoT devices. The possible threats are identified and prefetched to IoT gateways, which enables easy identification of malicious traffic in the data sources. Hence, the developed model is very effective in identifying malicious traffic, specifically scanning HTTP protocol at the port level. Additionally, it is designed to handle flood attacks with superior accuracy and a high positive rate.

In 2021, Alkadi *et al*. [20] suggested a Deep Blockchain Framework (DBF) to provide security-related distribution intrusion detection and privacy related to blockchain presented in IoT networks. In this framework, intrusion detection was performed with the help of Bidirectional Long Short-Term Memory (BiLSTM) along with deep learning approaches to perform effectively dealing with sequential network data. Additionally, blockchain models focusing on privacy and smart contract functionalities were developed, utilizing the Ethereum library to create an effective intrusion detection engine. The developed DBF model was contrasted with existing intrusion detection models and also their validation outcome was analysed. In 2022, Prajisha and Vasudevan [21]. Proposed an effective intrusion identification framework in an MQTT-based IoT network with the help of an improved Chaotic Salp Swarm Optimization Algorithm (ECSSA) and LightGBM classifier. Conventional IDS utilized multiple unwanted data as well as irrelevant attributes, which led to large time consumption and also reduced performance rate. To address these issues, developed ECSSA to offer effective identification in terms of accuracy. Experimental analysis performed on the developed model achieved an effective intrusion detection rate compared to the existing model.

In 2021, Fatani *et al*. [22] Recommended an effective IDS framework for IoT systems. They leveraged advancements in heuristic approaches and deep learning models to achieve high-performance rates in addressing challenging engineering issues. Here, a feature extraction approach was developed by utilizing CNN model for acquiring accurate features, and also a novel feature selection framework was developed by using Transient Search Optimization (TSO) with Differential Evolution (DE) and named (TSODE). In TSODE, the balancing processes were enhanced with the help of DE in the exploration and exploitation stages. As a result of these innovations, the suggested model secured an enhanced detection rate when contrasted over a conventional framework. In 2022, Basati and Mehdi [23] Proposed a novel lightweight framework with the help of a Parallel Deep Auto-Encoder (PDAE). The framework incorporated nearby data and locally available information in a feature vector, allowing researchers to enhance the accuracy rate in the developed model by minimizing parameters, processing power, and footprint memory. The efficacy of the developed model was validated with the help of different datasets, and it achieved a better rate in terms of performance as well as accuracy.

In 2021, Kan *et al*. [24] introduced a new IDS model according to Adaptive Particle Swarm Optimization with CNN (APSO-CNN). The model utilized the PSO approach with inertia weight to tune the parameters of a one-dimensional CNN. In the validation set, the value of the cross-entropy loss function was attained from CNN’s first training and it was considered as PSO fitness value. A novel validation scheme considered the probability of prediction allocated to several types, and also prediction label was contrasted over the developed APSO-CNN model. At the same time, validation measures were performed on the developed model with three different approaches over five conventional validation indicators. The validation outcome displayed that the suggested intrusion detection attack model was highly reliable and effective. In 2022, Breitenbacher *et al*. [25] proposed an efficient Host-based Anomaly Detection System for IoT (HADES-IoT). The suggested models have the ability to perform proactive detection and they also offered tamper-proof protection in IoT devices. The developed model has a reduced overhead rate and also it makes highly suitable for the system related to Linux in IoT devices. Thus, the developed model was analysed in seven different IoT devices and it achieved effective malware detection rates in IoT, IoT Reaper, and Mirai malware.

### 2.2 Problem statement

Nowadays, intrusion detection for IoT devices is highly essential due to several attacks performed in network systems. But, these types of systems had undergone different challenges like minimal detection rate, response time, unbalanced dataset, and also false alarm rate. Some of the existing approaches utilized to perform intrusion detection in IoT systems are showcased in Table 1. MQTT [18] is an effective approach to detect various attacks in the system and attained an enhanced accuracy rate than existing models. But it didn’t support highly advanced versions like flow control. Passban IDS [19] has the capability of securing both latter and IoT devices which are directly connected with it. It has the efficacy to train the system automatically by utilizing legitimate traffic flow in the target network. At the same time, it highly affects the data rate memory at the time of handling incoming traffic. DBF [20] offers a superior privacy preservation rate in a cloud environment. It also allows interchanging the data over a cloud sampler and effectively minimizing overhead. However, it needs to concentrate on utility as well as scalability to enhance the performance rate of the system. ECSSA [21] effectively reduces the computational time complexity and it has a better scalability rate also, they are easy to implement. Moreover, it has a very low convergence rate, and it slows down the process in local optima.

**Table 1:** Features and challenges of existing intrusion detection in IoT devices.

Author [citation]	Methodology	Features	Challenges
Siddharthan *et al*. [18]	MQTT	It effectively detects various attacks in the system and attained an enhanced accuracy rate than existing models.	It didn’t support highly advanced versions like flow control.
Eskandari *et al*. [19]	Passban IDS	It can secure the IoT devices that are directly connected to it. It has the efficacy to train the system automatically by utilizing legitimate traffic flow in the target network.	It highly affects the data rate memory at the time of handling incoming traffic.
Alkadi *et al*. [20]	DBF	It offered a superior privacy preservation rate in the cloud environment.	It needs to concentrate on utility as well as scalability for enhancing the performance rate of the system.
		It allows interchanging the data over a cloud sampler and effectively minimizing overhead.	
Prajisha and Vasudevan [21]	ECSSA	effectively reduces the computational time complexity, has a better scalability rate, and is also easy to implement	It has a very low convergence rate and its slowdown the process in local optima.
Fatani *et al*. [22]	TSODE	It enhances the searching process to avoid shortcomings attained in local optima.	It has a poor exploitation rate and it leads to several complexities in the system.
Basati and Mehdi [23]	PDAE	It enhances the accuracy rate by utilizing the minimum number of parameters.	It loses some important data at the time of processing and it creates multiple challenges in the system.
		It minimizes the noise effectively and offered an enhanced attack detection rate.	
Kan *et al*. [24]	APSO-CNN	It has the ability for predicting the state of the hacker’s upcoming attack.	It needs to minimize time complexity issues and need to analyze the attack stage.
Breitenbacher *et al*. [25]	HADES-IoT	It has very less number of overhead and offered effective attack classification rates.	It is highly complex to identify the attack type and challenging due to a huge number of parameters.
		It offered tamper-proof security to the system for a wide range.	

TSODE [22] enhances the searching process to avoid shortcomings attained in local optima. However, it has a poor exploitation rate and leads to several complexities in the system. PDAE [23] enhances the accuracy rate by utilizing a minimum number of parameters and it minimizes the noise effectively, offering enhanced attack detection rate. But it loses some important data at the time of processing and creates multiple challenges in the system. APSO-CNN [24] has the ability to predict the state of a hacker’s upcoming attack. At, the same time, it needs to minimize time complexity issues and need to analyze the attack stage. HADES-IoT [25] has a very less number of overhead and offers effective attack classification rates. It also offers tamper-proof security to the system for a wide range. But it is highly complex to identify the attack type and challenging due to a huge number of parameters. So, it is highly essential to develop a new automatic intrusion detection system in IoT devices.

## 3. Behavior and characteristics of the MQTTset and experimented dataset description

### 3.1 Background of MQTT set

IoT-Flock is an open-source tool used to develop the dataset and they are referred to as network traffic generator tools. To analyse IoT devices and also their network with the help of MQTT and CoAP protocol. Basically, IoT-Flock is surrounded by a network configuring environment related to node information like sensor type, listening ports, and IP addresses. Likewise, communication-related information such as time intervals is acquired to perform communication with the sensor.

**Fig 1:**
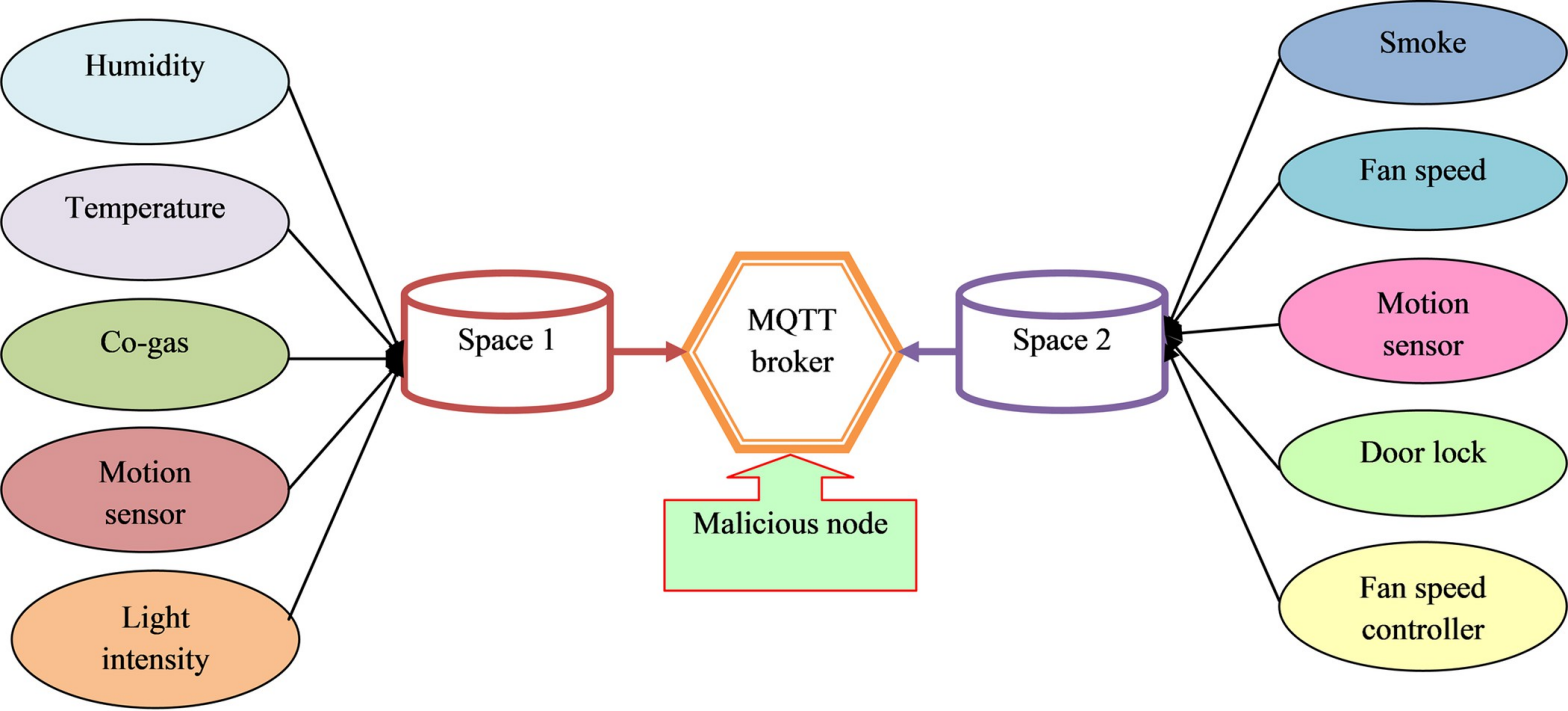
Architectural view of smart home atmosphere.

With respect to MQTT and CoAP protocol and the following attacks are listed as packet crafting attacks, memory leak attacks over CoAP, publish flood, and segmentation fault attacks over CoAP. These kinds of datasets are mostly used in environments of the real world where multiple IoT sensors are connected with multiple sensors. Smart homes are needed to consider this situation and sensors are established by utilizing IP addresses to rectify information such as humidity, fan status, intensity of light, motion, door opened/closed, CO-Gas, temperature, and smoke in different temperature circumferences. Architectural views of the smart home environment are presented in Fig 1.

In the initial phase, the sensor network is implemented in a limited access area for performing effective interaction with the broker with the help of sensors. They are mainly implemented without any additional elements, and traffic capturing is performed inside the broker. In the attack phase, malicious nodes are directly connected through the broker to execute a cyber-attack. Then, the entire communication characters associated with several sensors during this attack are displayed in Table 2. This table provides details on the periodic transmission of messages, as well as the random intervals at which messages are sent.

**Table 2:** Information of sensor nodes in IoT devices with MQTT set.

Sensor	Room	Messages Frequency	IP Address	Data Profile	Type	Topic
Smoke	2	3600	192.168.0.180	Smoke	Random	Smoke
Humidity	1	60	192.168.0.152	Humidity	Periodic	Humidity
Door lock	2	3600	192.168.0.176	Door lock	Random	Door lock
Motion sensor	1	3600	192.168.0.154	Movement	Random	Movement
Fan speed controller	2	120	192.168.0.173	Fan speed	Periodic	Fan speed
Light intensity	1	1800	192.168.0.150	Light intensity	Periodic	Light Intensity
Fan sensor	2	60	192.168.0.178	Fan	Periodic	Fan
CO-Gas	1	3600	192.168.0.155	CO-Gas	Random	CO-Gas
Temperature	1	60	192.168.0.151	Temperature	Periodic	Temperature
Motion sensor	2	3600	192.168.0.174	Movement	Random	Movement

### 3.2 Attacks in MQTT set

Cyber-attacks performed in the MQTT set are observed and they are included real attacks to target the required MQTT network for validating the dataset which is utilized to calculate the performance rate of the detection approaches. Here, the various types of attacks observed in MQTT set are thoroughly explained as follows.

#### 3.2.1 MQTT publish flood

At the time of transmission an enormous amount of MQTT set was over malicious IoT devices, this attack is suggested to contrast the source by utilizing a single connection without waiting for various connections.

#### 3.2.2 Brute force authentication

It has the capability to add running achievable attempts to regain the identification hired for MQTT.

#### 3.2.3 Malformed data

It mainly aimed to create and transfer the malformed packet to the broker which tries to enhance the exception against the targeted service.

#### 3.2.4 Flooding DoS

These kinds of attacks are mainly used to minimize the services offered to the client by regulating them. So, the MQTT protocols have appeared fixed in the destination to stature the broker by adding different connections over the broker.

#### 3.2.5 SlowITe

It is a new kind of DoS attack that is mainly developed to attack the applications of MQTT protocol and it required a minimal bandwidth consumption rate, as well as resources, needs to target the services of MQTT.

The information on sensor nodes in IoT with an MQTT set is shown in Table 3.

**Table 3:** Threats in MQTT set.

Attack	Number of Packets	Time (mm: ss)	PCAP Size (bytes)
MQTT Publish Flood	613	05:00	8212656
Brute Force Authentication	14501	30:00	1397132
SlowITe	9202	10:00	972, 272
Malformed Data	10924	06:00	1038590
Flooding Dos	130223	05:00	49875539

### 3.3 Details of experimented dataset

MQTT sets are used to concentrate on different characteristics of connection and acquired them from the raw data of the network. These kinds of elements are utilized to classify the potential attack as well as their authorized attack. Then, unnecessary characters are eliminated to perform intrusion detection and they are listed as follows MQTT topic, tcp.window_size, password, MQTT client Id, username, tcp.stream, communication times, Source or destination addresses, iRTT, and ports. The above-mentioned features are removed from the dataset, to acquire the appropriate data from the network for performing effective intrusion detection. The MQTT set consists of 34 features related to the MQTT protocol, they are tabulated in Table 4.

**Table 4:** Features of MQTT set.

S no	Features	Characteristics
1	tcp.flags	TCP packet transfers
2	tcp.time_delta	TCP Delta Time measures between the prior and current packet
3	tcp.len	TCP Header length
4	mqtt.conack.flags	MQTT CONNECT and response messages
5	mqtt.conack.flags.reserved	reserved flag in the CONNECT
6	mqtt.conack.flags.sp	Session present flag in the CONNECT
7	mqtt.conack.val	extracted the sequence data from the packet
8	mqtt.conflag.cleansess	Clean Session flag
9	mqtt.conflag.passwd	Password file Specified
10	mqtt.conflag.qos	Quality of service level
11	mqtt.conflag.reserved	Reserved
12	mqtt.conflag.retain	Will Retain
13	mqtt.conflag.uname	MQTT, User name flag.
14	mqtt.conflag.willflag	Will Flag
15	mqtt.conflags	Connection Flags
16	mqtt.dupflag	Duplicate Flags
17	mqtt.hdrflags	Indicates Header flags
18	mqtt.kalive	Keep Alive MQTT
19	mqtt.len	Message Length
20	mqtt.msg	Message
21	mqtt.msgid	Message ID from an incoming MQTT message
22	mqtt.msgtype	MQTT message type belonging
23	mqtt.proto_len	Protocol Name and length
24	mqtt.protoname	Protocol Name
25	mqtt.qos	Quality of service level (Qos 1, Qos 2, Qos 3)
26	mqtt.retain	The retain flag
27	mqtt.sub.qos	Request for Quality of Service
28	mqtt.suback.qos	Granted for Quality of Service
29	mqtt.ver	Version of MQTT
30	mqtt.willmsg	Will message
31	mqtt.willmsg_len	Length of will message
32	mqtt.willtopic	The topic of will message
33	mqtt.willtopic_len	The topic and length of will message
34	target	Provides the output

The following features are extrapolated and provided by the MQTT set. Such features were extracted for both legitimate and malicious cases.

In the model training process, all the mentioned features play crucial roles. During the preprocessing stage, essential steps such as data cleaning are performed to eliminate inconsistencies, and normalization is applied to standardize the data. Subsequently, any missing values (NaN) are replaced with zero, and statistical features like Maximum, Mean, Median, Standard deviation, and Variance are computed. Additionally, duplicate entries are removed using the df.drop_duplicates() function. To further enhance the data, the Min, Max, and Scaler are applied to scale the values appropriately.

The collected MQTT set to perform the efficient analysis is termed as $DA_{z}^{inp}$ and they are provided as the input for pre-processing phase. Here, the term *z* = 1,2,…,*Z* and the total number of data is given as *Z*.

### 3.4 Pre-processing of data

Initially, the acquired data $DA_{z}^{inp}$ from the MQTT set is used as the input for pre-processing phase. Here, pre-processing is performed with the help of data cleaning and transformation are performed. The major aim of pre-processing is to replace the nan values as well as to remove the duplicate data presented in the input data. Data cleaning is achieved to attain clean data to perform further processes and also data cleaning is referred to as a process that has the capability to fix corrupt, duplicate, incorrect, incomplete data and incorrectly formatted data presented in the dataset. Once, the data is cleaned then, data transformation is carried out on the cleaned data. Data transformation is defined as the process of modifying the structure, design, values, and format of data and also it can enhance the efficacy rate of the analytical process. Here, the pre-processed data are attained by utilizing data cleaning as well as data normalization, and they are indicated as $DA_{z}^{pre}$, which are further used in the feature extraction phase.

## 4. Architecture of proposed intrusion detection model in IoT using MQTTset with the meta-heuristic concept

### 4.1 Architecture of MQTT-based IoT intrusion detection

IoT is considered as enhancing technology that mainly connects different devices like smartphones, IP cameras, wireless sensor nodes, and household applications like fridges, TVs, and so on with the internet without human help. IoT is detected as a highly susceptible network that is attacked both externally and internally by attackers. The major goal of external attackers is to corrupt the entire system by utilizing an outside network. Similarly, internal attackers do violence to the system by inserting a threat inside the network. So, the internal attacker easily accesses the information of the network when compared with external attackers. Mostly, attackers collect different information about the system for checking the vulnerability with the help of various tools like shodan, Masscan, and Network Mapper (NMAP).

**Fig 2:**
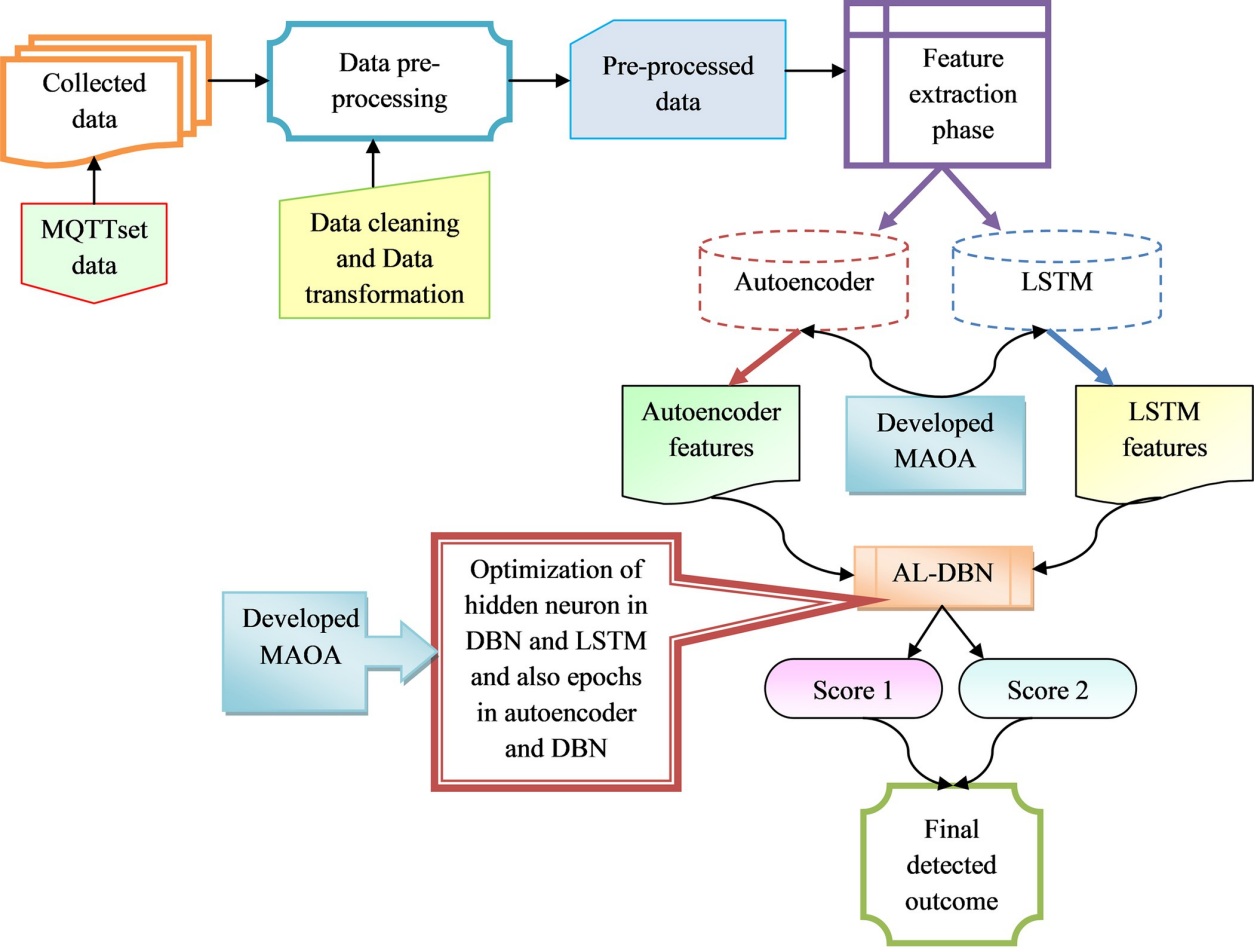
Architectural view developed intrusion detection model for IoT device.

Then, to offer secured and effective data exchange over IoT nodes, multiple protocols related to messaging and communication have been enhanced. Some of the developed protocols are MQTT, Advanced Message Queuing Protocol (AMQP), and Constrained Application Protocol (CoAP). In smart homes, industrial applications, and agricultural IoT, mostly MQTT is utilized because they highly support different communication with low bandwidth, minimized pocket loss, and minimal memory. However, this MQTT protocol did not have any kind of security settings and the major reason for this complication is the occupation of resources. IDS ensures the real-time monitoring of network activities, identifying and thwarting any unauthorized access or malicious activities. Additionally, strong authentication protocols provide an extra layer of security by verifying the identities of both clients and brokers, preventing unauthorized entities from exploiting vulnerabilities in MQTT communication. Commonly, Intrusion Detection Systems and authentication mechanisms are widely employed to safeguard the MQTT protocol, yielding effective outcomes even in complex scenarios. However, their extensive use is hindered by their inherent complexity, as they may be tailored for specific narrow conditions, posing challenges during implementation. Therefore, it becomes imperative to devise a new intrusion detection model tailored for IoT devices. The developed IDS are represented in Fig 2.

A novel intrusion detection model has been developed with the help of deep learning approaches for IoT devices. Initially, MQTT set was acquired from standard benchmark sources and offered as input to pre-processing phase. In this phase, the input data are pre-processed with the help of data cleaning and data transformation techniques.

The major aim for performing pre-processing is to replace the nan values and remove duplicate data that occurred in the network. Then, the pre-processed data are offered in the feature extraction phase. Here, two types of feature extraction have occurred which are autoencoder-based DBN features and LSTM-based DBN features.

The pre-processed data are offered as the input for the autoencoder-based feature extraction phase. The epochs of the autoencoder are optimized with the help of developed MAOA and secured autoencoder features. The attained autoencoder features are provided as the input for the DBN detection phase, and the DBN parameters like epochs and hidden neurons are tuned with the help of the developed MAOA. They secured the DBN score of 1. Further, the pre-processed data are offered as the input for the LSTM-based feature extraction phase and also the hidden neuron count of LSTM is tuned with the help of developed MAOA. The secured LSTM features are obtained. In the DBN detection phase, LSTM features are offered as the input and the parameters of DBN like hidden neuron count as well as epochs are optimized by utilizing developed MAOA to obtain the DBN score 2. The two different scores attained from AL-DBN are averaged and offer the final classified outcome. Finally, the rate of accuracy is enhanced to offer a highly accurate intrusion detection rate.

### 4.2 Proposed MAOA

A novel optimization approach named MAOA has been developed and implemented in the intrusion detection model to optimize different parameters like epochs count in autoencoder and DBN and also hidden neuron count in LSTM and DBN to offer an effective intrusion detection rate. AOA [26] has very low computation cost when contrasted with conventional approaches, and it has the ability to offer effective outcomes by resolving optimization issues. However, it has very low search and exploring capability and also it falls on the local optima phase. To overcome the above-listed complexities, a new scheme named MAOA is designed. The density decreasing factor is computed by Eq (1).


D=1−FT (1)

Here, the term *FT* denotes the transfer operator, and the term *D* indicate the density-decreasing factor.

AOA is an approach developed based on population, where the population individuals are considered objects. Like conventional approaches, AOA performs searching with the initial object population along with random densities, volumes, and acceleration. Initially, several objects are utilized with random populations in the fluid. AOA is a global optimization approach that contains two different phases exploitation as well as exploration.

#### Phase 1: Initialization

The positions of the entire object are initialized with the help of Eq (2).


$$C_{v}=ja_{v}+Rnd\times (ka_{v}-ja_{v});v=1,2,\ldots ,I$$ (2)

Here, the term *C*_*v*_ indicates *v*^*th*^ the population which is presented in *I* the object. The upper bound of the search space is given as *ka*_*v*_ and the lower bound of the search space is indicated as *ja*_*v*_. Then, initialization is performed for volume as well as density in several *v*^*th*^ objects and it is represented in Eq (3).


$$\begin{array}{l} Den_{v}=Rnd\\ Vol_{v}=Rnd \end{array}\}$$ (3)

Here, the term *Den*_*v*_ indicates the object density, *Vol*_*v*_ denote the volume of the object, and also *Rnd* is defined as the dimensional vector which randomly produces the random number in the range of [0,1]. The acceleration initialization for *v*^*th*^ the object is attained based on Eq (4).


$$Ace_{v}=ja_{v}+Rnd\times (ka_{v}-ja_{v})$$ (4)

Here, the term *Ace*_*v*_ denotes the acceleration of *v*^*th*^ the object. Then, validations are performed on the initial population and choose the object based on fitness value and determine the values of *Den*_*bst*_, *Ace*_*bst*_, *Vol*_*bst*_and *y*_*bst*_, respectively.

#### Phase 2-volume and density update

In this phase, the density as well as volume *v*^*th*^ object is updated for the iteration *s*+1 and they are updated by Eq (5).


$$\begin{array}{l} Den_{v}^{s+1}=Den_{v}^{s}+Rnd\times (Den_{bst}-Den_{v}^{s})\\ Vol_{v}^{s+1}=Vol_{v}^{s}+Rnd\times (Vol_{bst}-Vol_{v}^{s}) \end{array}\}$$ (5)

Here, the term *Vol*_*bst*_ denotes the volume of the best object and *Den*_*bst*_ denotes the density of the best term, and random numbers which are uniformly distributed are given as *Rnd*.

#### Phase 3-Density factor and transfer operation

Initially, collision is performed among the object after a certain time period both the object tries to secure the equilibrium phase. This condition is included in the AOA approach with the help of the transfer operator *FT* which has the capability to transfer the search from the phase exploration to exploitation and it is indicated in Eq (6).


$$FT=\exp(\frac{v-v_{mx}}{v_{mx}})$$ (6)

Here, the term *v* denotes the iteration number and *v*_*mx*_ indicate the maximum iteration. The term *FT* improved slowly until it reaches condition 1. Likewise, decreasing density factor *D* helps AOA in global and local search that is formulated adaptively with the transfer operator in the proposed MAOA, but in the conventional algorithm, it is considered as the random variable. The timing is minimized by utilizing Eq (7).


$$D^{v+1}=\exp(\frac{v_{mx}-v}{v_{mx}})-(\frac{v}{v_{mx}})$$ (7)

Here, the term *D*^*v*+1^ minimized with time and offered the capability for meeting the already detected area.

#### Phase 4.1-Exploration

In this phase, collision is occurring between two different objects. If it satisfies the condition *FT*≤0.5, then a collision is attained between the object. Then, random material as well as object acceleration is attained for iteration *v*+1 with the help of Eq (8).


$$Ace_{s}^{v+1}=\frac{Den_{za}+Vol_{za}\times Ace_{za}}{Den_{s}^{v+1}\times Vol_{s}^{v+1}}$$ (8)

Here, the random material is termed as *za*, the object acceleration for *s*^*th*^ the object is indicated as *Ace*_*s*_, *Vol*_*s*_, *Den*_*s*_ and also density, volume, and acceleration for random material are given as *Den*_*za*_, *Vol*_*za*_, *Ace*_*za*_, respectively. Then, *FT*≤0.5 allow exploration when it achieves one-third of iteration and also applies the value other than 0.5 and shift from the exploration stage to the exploitation stage.

#### Phase 4.2-Exploitation

In this phase, no collision occurred between the object for the condition *FT*>0.5 and the acceleration object is updated for the iteration *v*+1 by utilizing Eq (9).


$$Ace_{s}^{v+1}=\frac{Den_{bst}+Vol_{bst}\times Ace_{bst}}{Den_{s}^{v+1}\times Vol_{s}^{v+1}}$$ (9)

Here, the term *Ace*_*bst*_ denotes the best object acceleration.

#### Phase 4.3-Acceleration normalization

Normalize the acceleration value to validate the percentage variation by utilizing Eq (10).


$$Ace_{s-norm}^{v+1}=y\times \frac{Ace_{s}^{v+1}-Mn(Ace)}{Mx(Ace)-Mn(Ace)}+x$$ (10)

Here, the range of normalization is indicated as *y*, *x*, and their value is fixed as 0.9 and 0.1, respectively. The term $Ace_{s-norm}^{v+1}$ indicates the step percentage of the entire agent. If the object value *s* is away from the global optimum, then the value of acceleration is comparatively high and the object has remained in the exploration phase otherwise the object presented in the exploitation phase. Normally, the acceleration factor starts from a large number and minimized with time and it helps the search agent to travel over the global best solution but they are destructed from the local solution in the normal phase. So, AOA attained a balanced state between exploration and exploitation.

#### Phase 5-Position updating

If the condition *FT*≤0.5 is fulfilled then the position of *s*^*th*^ an object is updated for upcoming iteration *v*+1 with the help of Eq (11).


$$g_{s}^{v+1}=g_{s}^{v}+W_{1}\times rnd\times Ace_{s-norm}^{v+1}\times D\times (g_{rnd}-g_{s}^{v})$$ (11)

Here, the term *W*_1_ denotes the constant and it is equal to 2 and the term *D* is updated by Eq (1). For the satisfaction of the condition *FT*>0.5 then, object positions are updated by utilizing the Eq (12).


$$g_{s}^{v+1}=g_{bst}^{v}+Fg\times W_{2}\times rnd\times Ace_{s-norm}^{v+1}\times D\times (Ti\times g_{bst}-g_{s}^{v})$$ (12)

Here, the term *W*_2_ indicates the constant which equals to the number 6, and the term *D* is updated by Eq (1). The term *Ti* enhanced along with time and they are directly propositional to *FT* and are equated as *Ti* = *W*_3_×*FT*. The term *Ti* enhanced with the time range as [*W*_3_×0.3,1] and the percentage is attained from the best position. The term *Fg* is the flag that is used to modify the motion direction by utilizing Eq (13).


$$Fg=\{\begin{array}{cc} +1 & if\;P\leq 0.5\\ -1 & if\;P>0.5 \end{array}$$ (13)

Here, the term *P* is given as *P* = 2×*rnd*−*W*_4_.

#### Phase 5-Validation

Validate the values of several objects by utilizing the objective function and also retain the best solution and allocates *I*_*bst*_, *Ace*_*bst*_, *Vol*_*bst*_ and *Den*_*bst*_. The pseudo-code for the developed MAOA is represented by Algorithm 1.

 **Algorithm 1: Developed MAOA**

Initialize the random population

Validate the initial population

Select the best fitness value

Set the iteration count *v* = 1

While *v*≤*v*_*mx*_ do

 For entire object *s* do

  Upgrade several object volume and density by Eq (5)

  Upgrade transfer factor by Eq (6)

  **Upgrade density decreasing factor *D* by Eq (1)**

  If *FT*≤0.5 then, exploration phase

   Upgrade acceleration by Eq (8)

   Upgrade normalize acceleration by Eq (10)

   Position update by Eq (11)

  Else reach exploration phase

   Acceleration update by Eq (9)

   normalized acceleration upgrade by Eq (10)

   Direction flag by Eq (13)

   Position update by Eq (12)

  End if

 End for

 Validate the object and choose best fitness value

End while

Return optimal best solution

**Fig 3:**
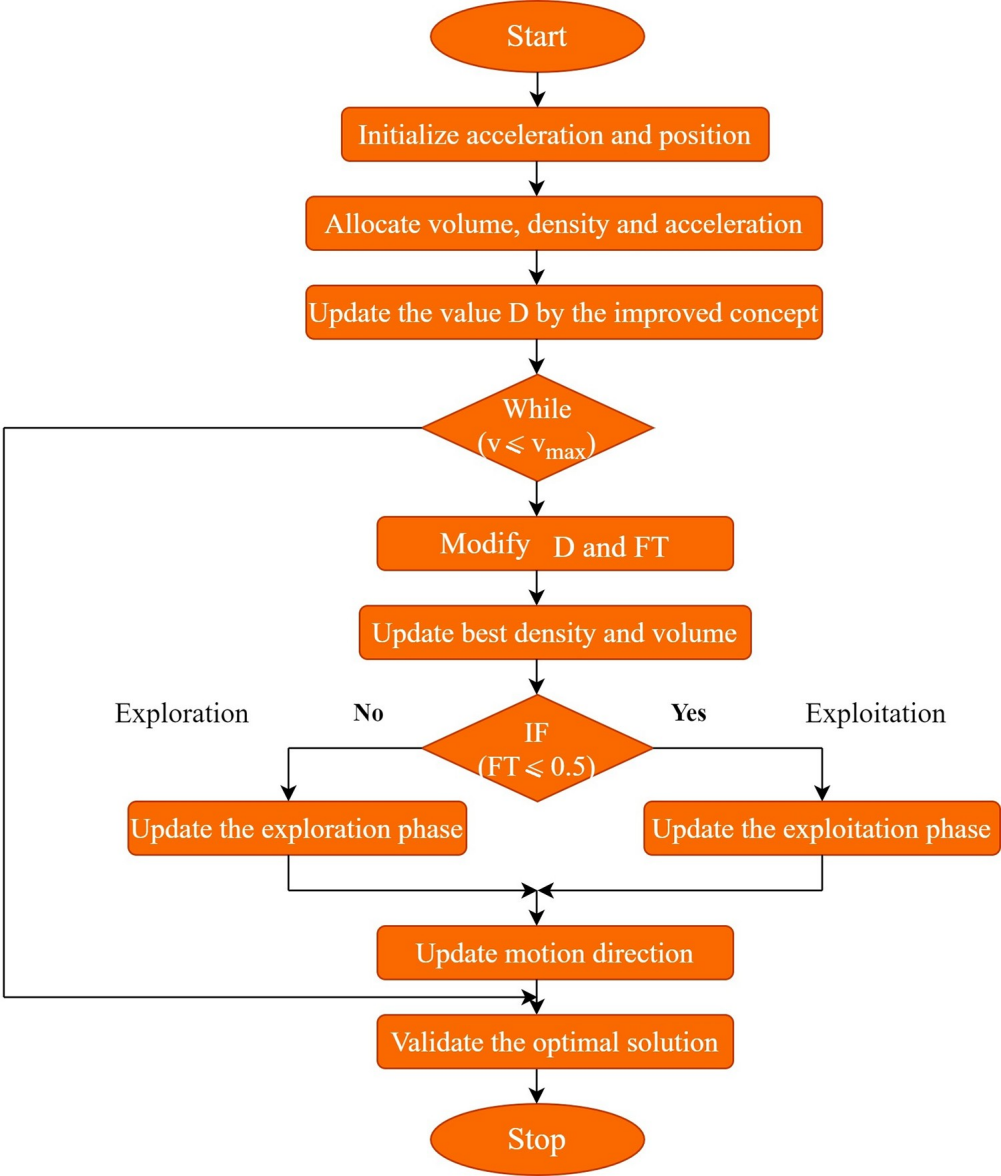
Flowchart for developed MAOA.

The flowchart for the developed MAOA is represented in Fig 3.

## 5. Development of autoencoder and LSTM-based Deep Belief Network for improved IoT intrusion detection

### 5.1 Model 1: Autoencoder with Deep Belief Network

The pre-processed data $DA_{z}^{pre}$ is provided as the input for the autoencoder to select the deep features from the feature extraction phase. Autoencoder [27], as well as CNN, share the same framework design in a network system, and also these models have similar features such as convolution filter, pooling layer, and basic component. In this architecture, the input node and the outcome node have similar measurements and it is considered as an essential character. But, when the learning process is performed, the system offered different information about the node, and they are not labeled as a dependent system. Moreover, the autoencoder structure didn’t pursue any special approach to performing the process. Thus, the feature map of *b*^*th*^ the inactive layer represented is offered in Eq (14).


$$f^{b}=\sigma (w*H^{b}+x^{b})$$ (14)

Here, the input attained from mono-channel is indicated as *w*, convolution for two-dimensional is given as *, and activation function is denoted as σ. Then, reconstruction performed in the decoder is achieved by Eq (15).


$$z=\sigma (\sum_{b\in J}f^{b}*{\tilde{H}}^{b}+i)$$ (15)

Latent feature map for the group is given as *J* and bias per input channel is indicated as *i*, respectively.

The acquired features from the autoencoder are indicated as $FE_{h}^{auto}$ and they are offered to the DBN network. DBN [28] is considered as a productive Deep Neural Network (DNN) framework. The Restricted Boltzmann Machine (RBM) is an integral component of the Deep Belief Network (DBN) framework. The DBN is a productive Deep Neural Network (DNN) architecture that combines a stacked RBM along with a sigmoid belief network. The DBN model utilized in this study consists of three different stacked Restricted Boltzmann Machines (RBM), denoted as RBM-1, RBM-2, and RBM-3. Additionally, it incorporates three hidden layers represented as A1, A2, and A3, respectively. The input layer is denoted as *Q* = *A*_0_, and connected to the first hidden layer A1, which represents the productive artificial neural network (ANN) that functions as RBM-1. During the training phase of the first layer, DBN employs a single layer with RBM-1, trained using the constructive divergence approach. In the second training phase, DBN adds two different layers to its structure. The upper layer is referred to as RBM-2, while the lower layer is termed SBN. During this phase, the weights of *C*_1_

SBN (lower layer) are frozen. Similarly, in the third training phase, DBN incorporates another layer, where the weights of *C*_1_, *C*_2_ RBM-3 are fixed. The mathematical representation of the DBN is represented in Eq (16), outlining the entire architecture and training process of the network. The acquired features from the autoencoder are passed on to this DBN network, contributing to the overall performance of the intrusion detection system.


$$R\{Y,A^{1},A^{2},\ldots ;A^{m}\}=R(Y|A^{1})R(A^{1}|A^{2})\ldots R(A^{\left(m-1\right)}|A^{\left(m\right)})$$ (16)

The probability rate *R*(*A*^(*m*−1)^|*A*^(*m*)^) is performed with RBM with Eq (17) and Eq (18).


$$R(A^{j}|A^{\left(j+1\right)})=\prod_{k}R(A_{k}^{j}|A^{\left(j+1\right)})$$ (17)

$$R(A_{k}^{j}|A^{\left(j+1\right)})=\sigma (h_{k}^{j}+\sum_{n}^{j+1}W_{nk}^{j}A_{n}^{j+1})$$ (18)

**Fig 4:**
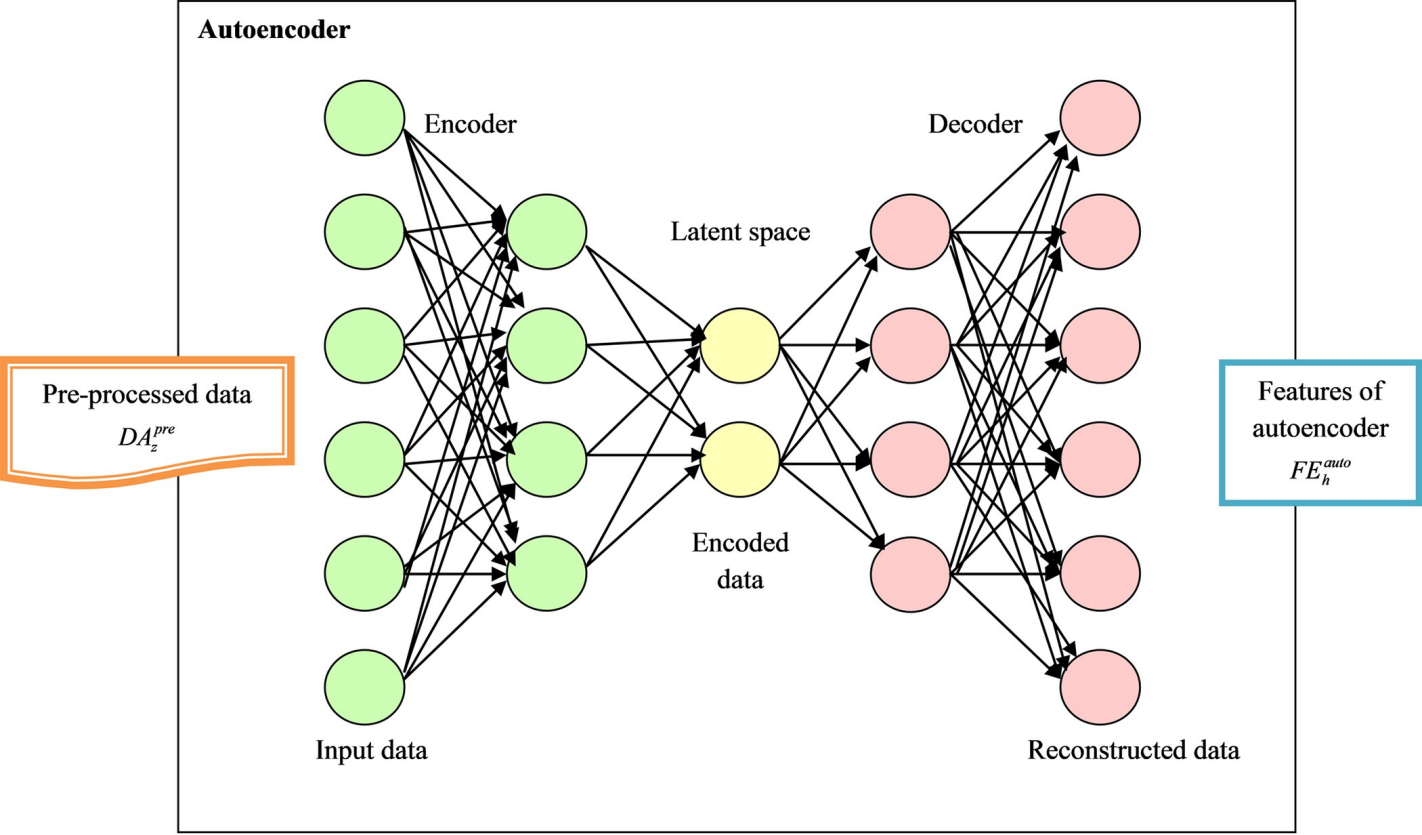
Representation of autoencoder with DBN.

Here, the greedy training model is utilized to train the RBM presented in DBN and also the RBM has the capability to develop features as well as reconstruct the input data. The structural view of the autoencoder with DBN is presented in Fig 4.

### 5.2 Model 2: LSTM with Deep Belief Network

The pre-processed data $DA_{z}^{pre}$ is provided as the input to LSTM to attain deep features from the feature extraction phase. LSTM [29] is considered as a unique Recurrent Neural Network (RNN) and it has the capability to offer highly precise time series-based prediction outcomes. In this LSTM framework, data information of existing neurons is linked with current neurons with the help of the LSTM unit. Then, major contractions are attained in-between LSTM and RNN in their memory cell. Later, long-term sequence predictions are improved by utilizing three multiple gates such as forget gate, output gate, and input gate. The input gate generates input by considering the new data which needs to recollect in the cell state. In forget state, it effectively eliminates the unused data stored in the cell state. So, finally, the output gate generates the data which is acquired in the cell state and the input gate is represented in Eq (19).


$$k_{u}=\sigma (M_{c}.[l_{u-1},q_{u}]+g_{c})$$ (19)

In Eq (20), the equation for forget gate is presented.


$$j_{u}=\sigma (M_{p}.[l_{u-1},q_{u}]+g_{p})$$ (20)

Units of LSTM are represented in Eq (21), Eq (22) and Eq (23).


$${\bar{y}}_{u}=\tan l(M_{i}.[l_{u-1},q_{u}]+g_{i})$$ (21)

$$y_{u}=j_{u}\times y_{u-1}+k_{u}\times {\bar{y}}_{u}$$ (22)

$$l_{u}=o_{u}\times \tan l(y_{u})$$ (23)

In Eq (24), the equation for the output gate is represented.


$$o_{u}=\sigma (M_{p}.[l_{u-1},q_{u}]+g_{z})$$ (24)

**Fig 5:**
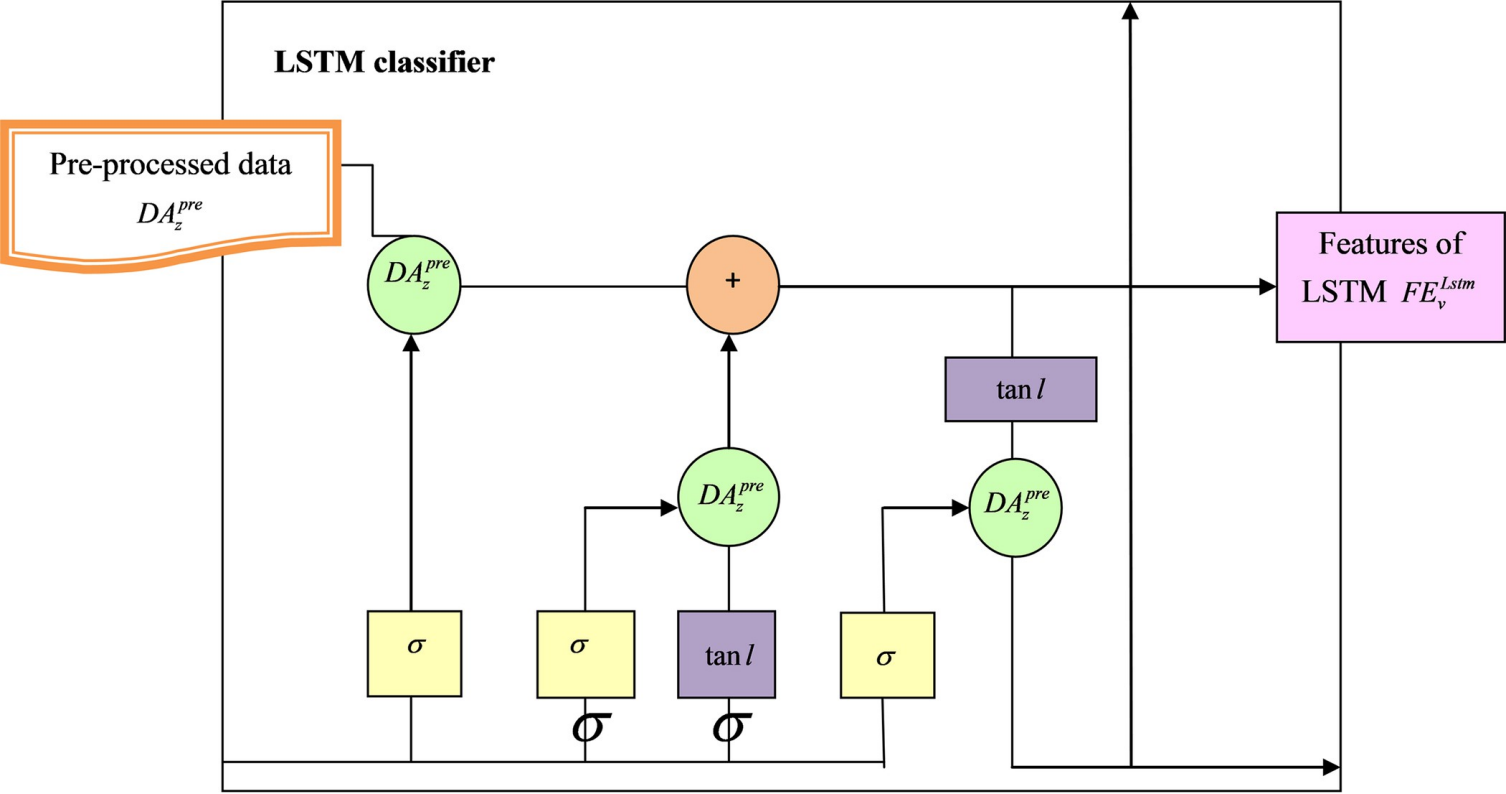
Representation of LSTM with DBN-based intrusion detection approach.

Here, the weight is denoted as *M*, bias attained in the training model is given as *g*, hidden states are indicated as *l*, and the cell state are represented as *y*. Finally, LSTM can resolve several gradient vanishing issues attained in conventional RNN structures. The acquired features from LSTM are represented as $FE_{v}^{Lstm}$ and they are offered to the DBN network and attained score 2. The diagrammatic presentation of LSTM-based DBN is presented in Fig 5.

### 5.3 Proposed AL-DBN with network optimization

Combination of Autoencoder and LSTM: The combination of autoencoding and LSTM in an intrusion detection system can result in a more robust and accurate system. Autoencoder helps in learning informative representations from the IoT data, while LSTM takes advantage of these representations to model the sequential patterns and detect anomalies. This combination enables the classifier to capture both the inherent structure and temporal dynamics of the IoT data. The resulting intrusion detection system is more robust and accurate.

The major aim of the developed AL-DBN is to offer an efficient intrusion detection rate in IoT devices. Autoencoders are mainly utilized to minimize the noise presented in the data and also to minimize the input size. But they are not effective to reconstruct the data and also it has a low number of training data. So, to tackle the above-mentioned complexities an effective LSTM is utilized. LSTM offered enhanced learning percentages in terms of learning rate and they are highly suitable for detection purposes.

**Fig 6:**
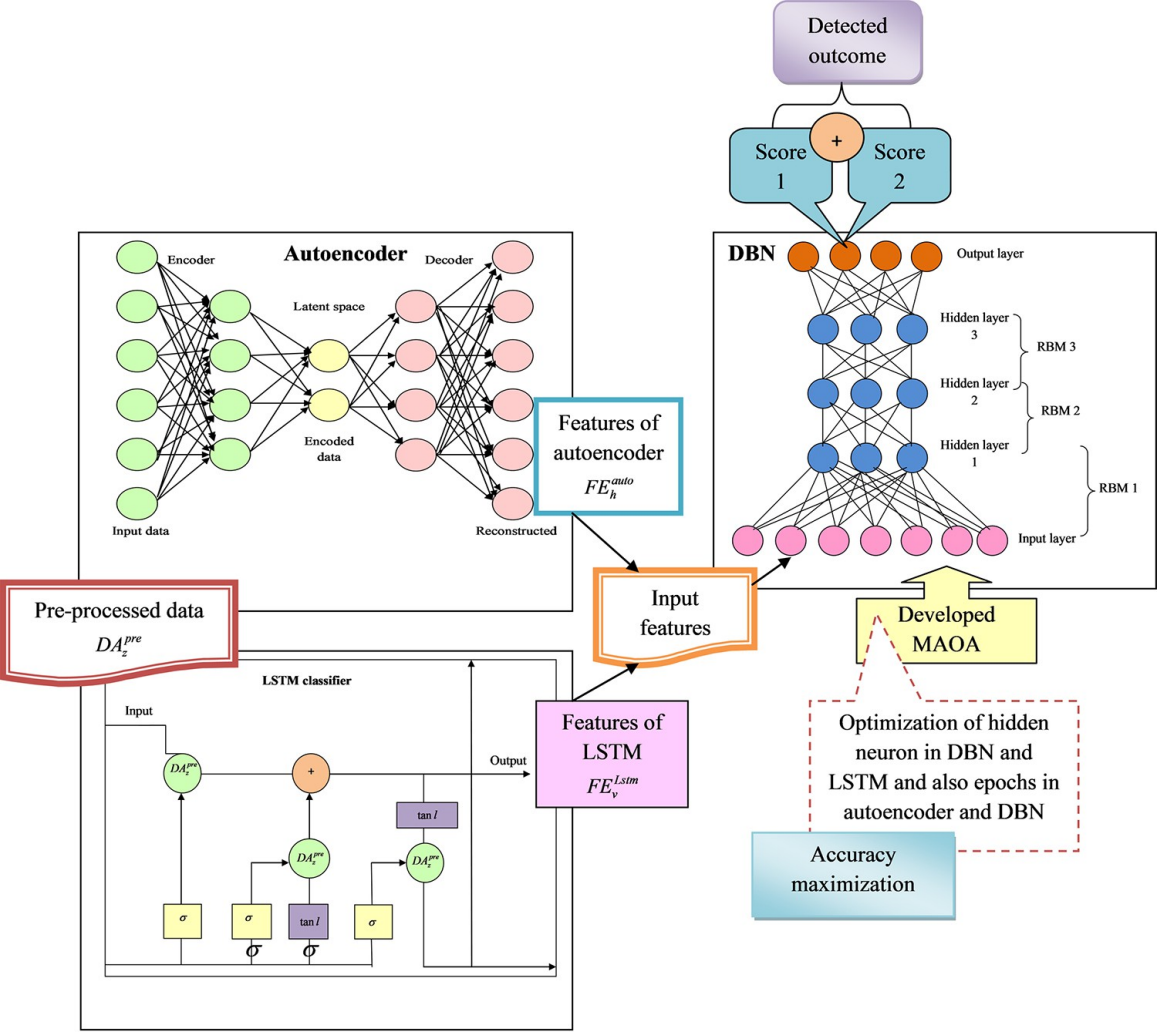
Structural view of developed AL-DBN-based intrusion detection architecture in IoT devices.

Thus, to secure an effective intrusion detection rate in IoT devices a novel fusion of AL-DBN is utilized. The diagrammatic presentation developed AL-DBN-based IDS is presented in Fig 6.

The developed AL-DBN offered two different scores, score 1 and score 2 which are attained when feature selection is performed in deep features. These two scores are averaged to attain the final intrusion detection rate along with an enhanced accuracy rate. Different parameters such as number of epochs in autoencoder and DBN as well as hidden neutron count in LSTM and DBN are tuned by utilizing the proposed MAOA among different tuning limits. The epochs of autoencoder and DBN are tuned in the range of [50,100] and also hidden neuron count in LSTM and DBN are tuned in the range of [5,255]. The fitness function for the suggested AL-DBN-based intrusion detection model is given in Eq (25).


$$Ft=\underset{\left\{HN_{t}^{LSTM},HN_{s}^{auto},EP_{q}^{LSTM},EP_{k}^{auto}\right\}}{\arg\max}(ACy)$$ (25)

Here, the term $HN_{t}^{LSTM}$ indicates the hidden neurons of LSTM, $HN_{s}^{auto}$ denote the hidden neurons of autoencoder, $EP_{q}^{LSTM}$ refers to the epochs count in LSTM, and $EP_{k}^{auto}$ represents the epochs of the autoencoder. The term *ACy* indicates accuracy and it is defined as validation of closeness for a specific value and is represented in Eq (26).


$$ACy=\frac{\left(yb+yc\right)}{\left(yb+yc+yd+ye\right)}$$ (26)

Here, the term *yb* denotes the true positive values, *yc* indicate the true negative value, *yd* determine the false positive value, and *ye* represents the false negative value.

## 6. Results and discussions

### 6.1 Experimental results

A novel intrusion detection model for IoT devices was implemented in the Python platform and also different analyses were performed to validate their efficacy rate over conventional classifiers and algorithms. In this analysis, the effective performed rate is attained with the help of maximum iteration 25 and population rate 10. Multiple contrasting approaches like Sunflower Optimization (SFO) [30], Jaya (JA) [31], Elephant Herding Optimization (EHO) [32], and AOA [26] along with classifiers like Random Forest with Support Vector Machine (RF+SVM) [33], DBN [28], LSTM [29], AL-DBN [34] were used for the calculation.

### 6.2 Performance metrics

The developed intrusion detection model for IoT devices is validated with several quantitative measures.

(a) Specificity (*spc*) is termed as the portion of negative value identified correctly and denoted in Eq (27).

$$spc=\frac{yc}{yc+yd}$$ (27)

(b) Sensitivity (*DB*) is determined as the positive ratio that are find out exactly and given in Eq (28)

$$DB=\frac{yb}{yb+ye}$$ (28)

(c) MCC (*JF*) is referred to as the binary quality analysis categorization and it is provided in Eq (29).

$$JF=\frac{yb\times yc-yd\times ye}{\sqrt{\left(yb+yd)(yb+ye)(yc+yb)(yc+ye\right)}}$$ (29)

(d) F1-score (*KS*) is termed as the accuracy calculation outcome and it is represented in Eq (30)

$$KS=2\times \frac{2yb}{2yb+yd+ye}$$ (30)

(e) FNR (*NT*) is described as the portion of positive occurred negative analysis outcome and it is offered in Eq (31).

$$NT=\frac{ye}{ye+yb}$$ (31)

(f) FPR (*RE*)is mentioned as a fraction between negative events and they are categorized wrongly as positive. So, the whole commonly available negative event is termed in Eq (32).

$$RE=\frac{yd}{yd+yc}$$ (32)

(g) NPV (*YW*) is referred to as the sum of whole individuals which are presented without any kind of defect when time analysis is achieved and they are represented in Eq (33).

$$YW=\frac{yc}{yc+ye}$$ (33)

### 6.3 Evaluation of the developed model with multiple classifiers and algorithms

Employing multiple deep learning models in the limited resources IoT environment offers benefits such as enhanced accuracy, robustness, feature complementarity, resource optimization, and adaptability. These advantages contribute to the overall effectiveness and efficiency of intrusion detection in IoT systems, improving the security posture of connected devices and networks.

**Fig 7:**
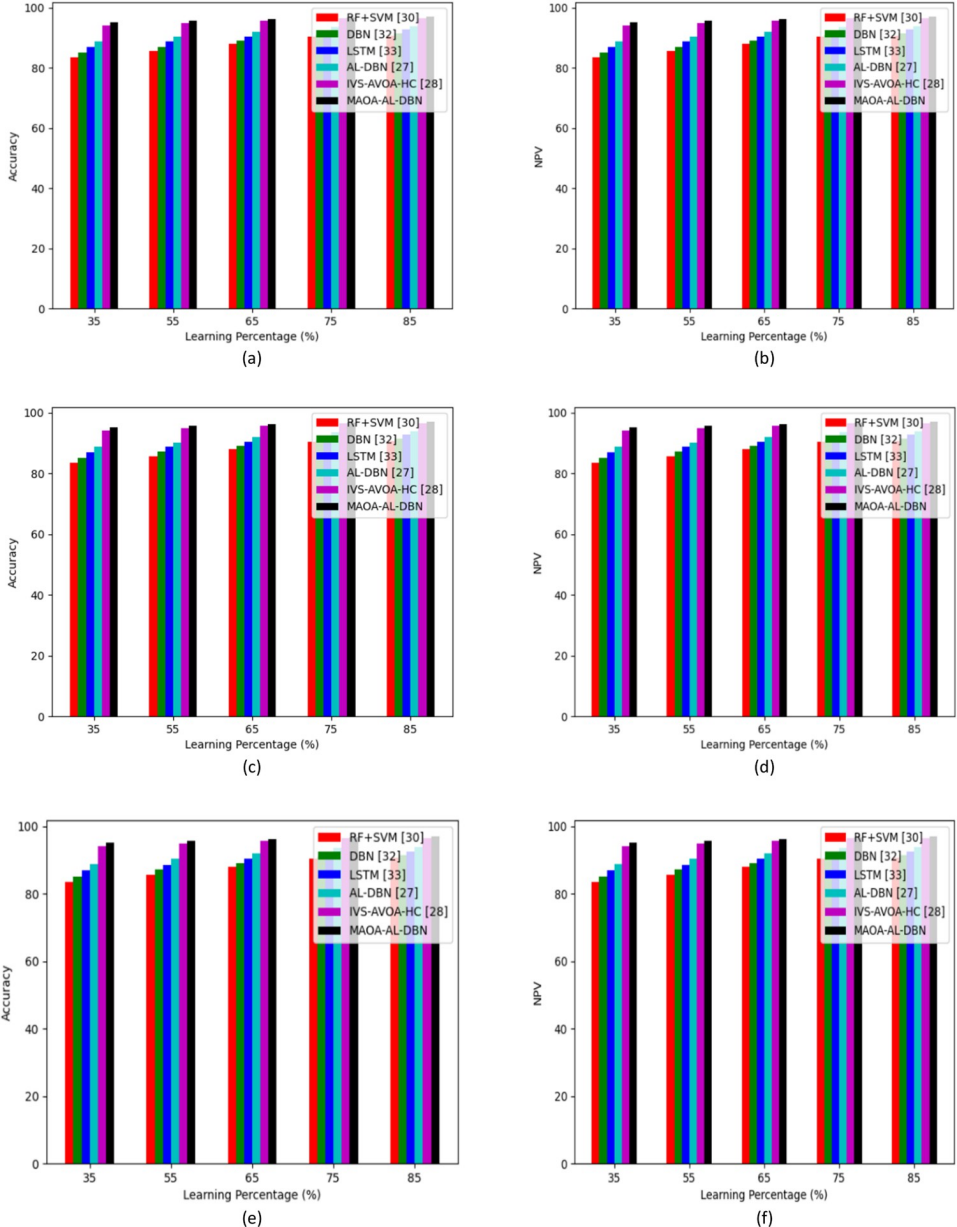
Analysis of developed intrusion detection model with multiple classifiers over (a) accuracy of test case 1 (b) NPV of test case 1(c) accuracy of test case 2 (d) NPV of test case 2 (e) accuracy of test case 3 and (f) NPV of test case 3.

**Fig 8:**
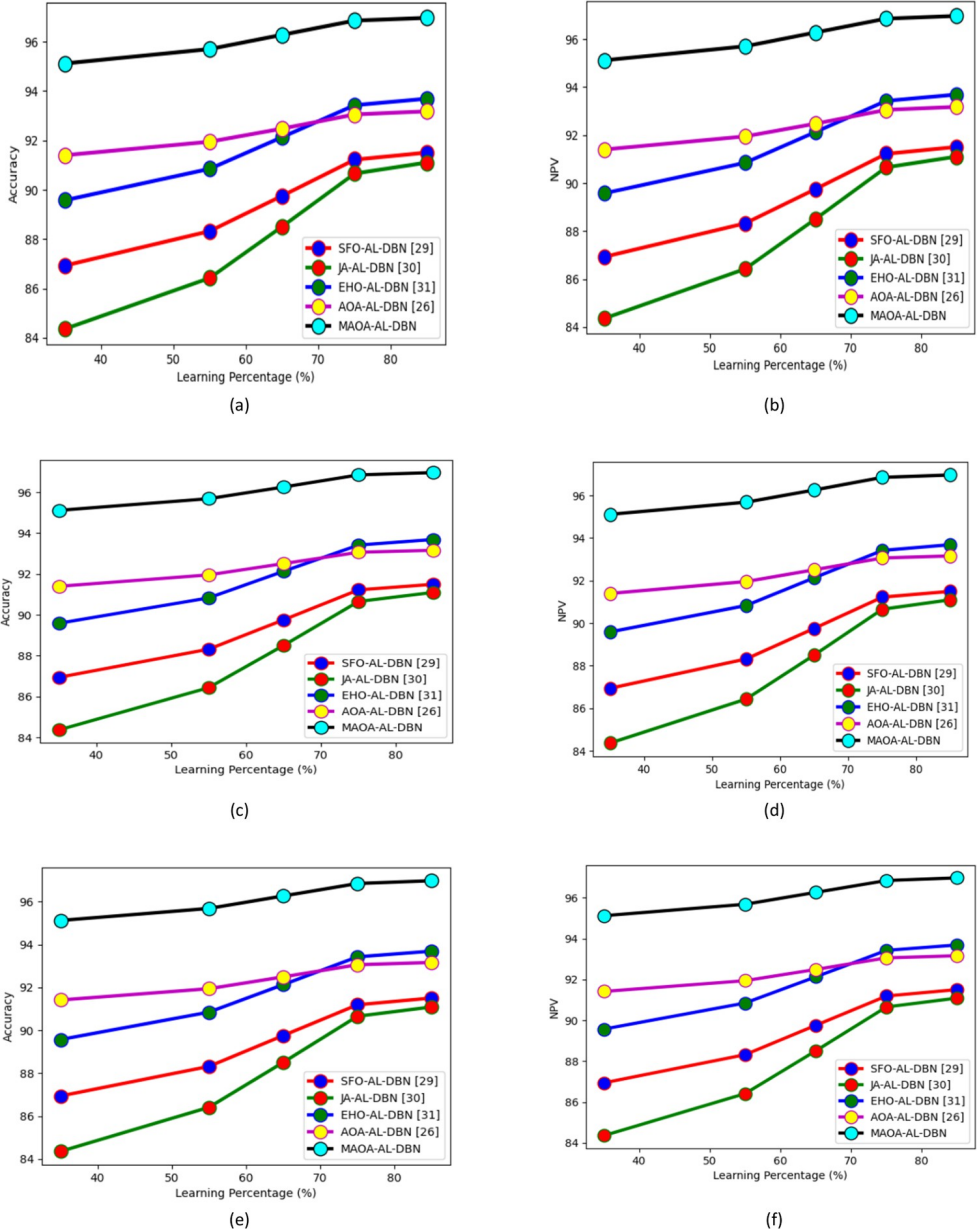
Analysis of developed intrusion detection model with several approaches over (a) accuracy of test case 1 (b) NPV of test case 1(c) accuracy of test case 2 (d) NPV of test case 2 (e) accuracy of test case 3 and (f) NPV of test case 3.

Different kind of analysis performed on classifiers and approaches using the developed MAOA-AL-DBN-base intrusion detection in IoT devices are represented in Figs 7 and 8. In the accuracy analysis performed 1^st^ test case of the developed intrusion detection model secured 10.3% enhanced than RF+SVM, 12.1% better than DBN, 11.26% greater than LSTM, 10.75% superior to AL-DBN and 6.7% improved than IVS-AVOA-HC in the learning percentage 65. Similarly, NVP analysis performed on conventional approaches, the developed model secured 8.16%, 10.54%, 5.2% and 3.24% higher than SFO-AL-DBN, JA-AL-DBN, EHO-AL-DBN and AOA-AL-DBN, respectively in learning percentage 55. Thus, the suggested intrusion detection model achieved significantly better performance rates than existing intrusion detection approaches.

### 6.4 K-fold analysis of the developed model with several classifiers and approaches

**Fig 9:**
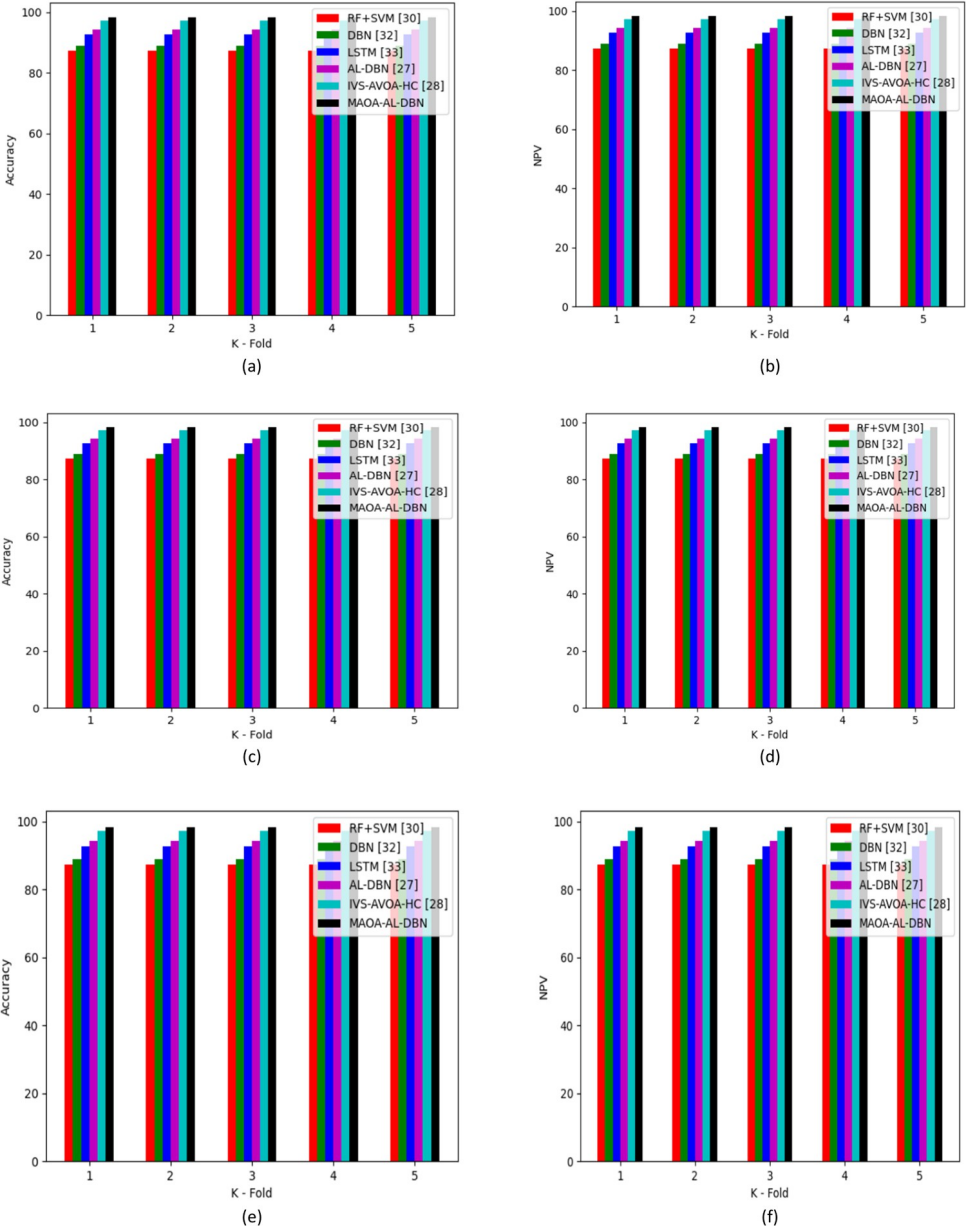
K-fold analysis on developed intrusion detection model with several classifiers over (a) accuracy of test case 1 (b) NPV of test case 1(c) accuracy of test case 2 (d) NPV of test case 2 (e) accuracy of test case 3 and (f) NPV of test case 3.

**Fig 10:**
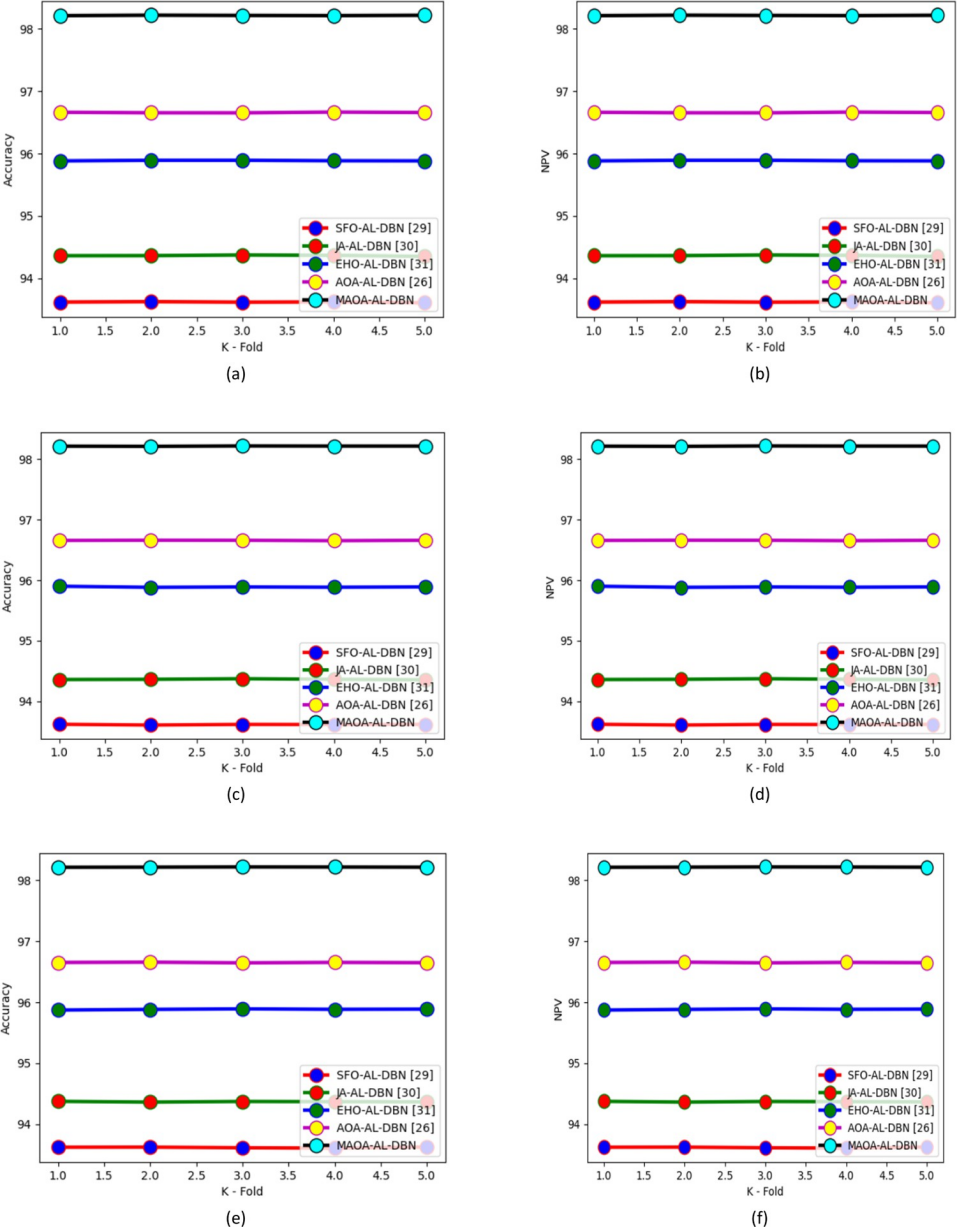
K-fold analysis on developed intrusion detection model with several approaches over (a) accuracy of test case 1 (b) NPV of test case 1(c) accuracy of test case 2 (d) NPV of test case 2 (e) accuracy of test case 3 and (f) NPV of test case 3.

In Figs 9 and 10 various observations performed on the suggested MAOA-AL-DBN-based intrusion detection model along with several classifiers and approaches are showcased. The developed model MAOA-AL-DBN secured highly accurate intrusion detection rates for various classifiers like RF+SVM, DBN, LSTM, AL-DBN, and IVS-AVOA-HC in the rate of 7.7%, 6.67%, 4.6%, 2.20%, and 1.45%, in 3^rd^ fold. Similarly, NPV analysis performed on the developed model achieved 5.4%, 4.2%, 2.9%, and 1.9% higher than SFO-AL-DBN, JA-AL-DBN, EHO-AL-DBN, and AOA-AL-DBN, respectively in 2^nd^ fold. Thus, this analysis displayed that the suggested model performed well than existing models and also detect the multiple intrusion in IoT devices.

### 6.5 Overall evaluation of algorithms and classifier on the developed model

Overall analysis performed on the developed MAOA-AL-DBN-based intrusion detection model in IoT devices are represented in Tables 5 and 6 with several heuristic approaches and classifiers.

**Table 5:** Evaluation of developed intrusion detection model with different heuristic algorithms.

Measures	SFO-AL-DBN [30]	JA-AL-DBN [31]	EHO-AL-DBN [32]	AOA-AL-DBN [26]	MAOA-AL-DBN
Test case 1					
Accuracy	91.50767	91.10567	93.69	93.177	**96.962**
Sensitivity	100	100	100	100	**100**
Specificity	91.50753	91.10552	93.68989	93.17689	**96.96195**
Precision	0.019622	0.018735	0.026406	0.024421	**0.054831**
FPR	8.492475	8.894482	6.310105	6.823114	**3.038051**
FNR	0	0	0	0	**0**
NPV	91.50753	91.10552	93.68989	93.17689	**96.96195**
FDR	99.98038	99.98126	99.97359	99.97558	**99.94517**
F1-Score	0.039236	0.037463	0.052798	0.048831	**0.109601**
MCC	0.0134	0.013065	0.015729	0.015085	**0.023057**
**Test case 2**					
Accuracy	91.49833	91.10233	93.691	93.16233	**96.96567**
Sensitivity	83.33333	83.33333	100	100	**100**
Specificity	91.4985	91.10249	93.69087	93.1622	**96.96561**
Precision	0.019601	0.018729	0.031691	0.029241	**0.065869**
FPR	8.501503	8.897511	6.309126	6.837803	**3.034394**
FNR	16.66667	16.66667	0	0	**0**
NPV	91.4985	91.10249	93.69087	93.1622	**96.96561**
FDR	99.9804	99.98127	99.96831	99.97076	**99.93413**
F1-Score	0.039193	0.037449	0.063361	0.058465	**0.131651**
MCC	0.011998	0.011691	0.017231	0.016505	**0.025273**
**Test case 3**					
Accuracy	91.51	91.093	93.69433	93.16733	**96.97933**
Sensitivity	100	100	100	100	**100**
Specificity	91.50986	91.09285	93.69423	93.16722	**96.97928**
Precision	0.019627	0.018708	0.026424	0.024387	**0.055145**
FPR	8.490142	8.907148	6.305772	6.832781	**3.020717**
FNR	0	0	0	0	**0**
NPV	91.50986	91.09285	93.69423	93.16722	**96.97928**
FDR	99.98037	99.98129	99.97358	99.97561	**99.94485**
F1-Score	0.039246	0.03741	0.052835	0.048761	**0.110229**
MCC	0.013402	0.013054	0.015735	0.015073	**0.023126**

**Table 6:** Evaluation of proposed intrusion detection model in IoT devices with several existing classifiers.

Measures	SVM [33]	DBN [28]	LSTM [29]	AL-DBN [34]	IVS-AVOA-HC [35]	MAOA-AL-DBN
**Test case 1**						
Accuracy	90.78333	91.43567	92.69167	93.94433	96.61803	96.962
Sensitivity	100	100	100	100	100	100
Specificity	90.78318	91.43552	92.69154	93.94423	96.61802	96.96195
Precision	0.01808	0.019457	0.0228	0.027515	0.001478	0.054831
FPR	9.21682	8.564476	7.308455	6.055768	3.381977	3.038051
FNR	0	0	0	0	0	0
NPV	90.78318	91.43552	92.69154	93.94423	96.61802	96.96195
FDR	99.98192	99.98054	99.9772	99.97249	99.99852	99.94517
F1-Score	0.036153	0.038906	0.045589	0.055015	0.002957	0.109601
MCC	0.012812	0.013338	0.014537	0.016078	0.003779	0.023057
**Test case 2**						
Accuracy	90.79233	91.44067	92.67733	93.94467	96.61707	96.96567
Sensitivity	83.33333	83.33333	100	100	100	100
Specificity	90.79248	91.44083	92.67719	93.94455	96.61706	96.96561
Precision	0.018098	0.019469	0.027305	0.033018	0.001478	0.065869
FPR	9.207517	8.559171	7.322813	6.055454	3.382935	3.034394
FNR	16.66667	16.66667	0	0	0	0
NPV	90.79248	91.44083	92.67719	93.94455	96.61706	96.96561
FDR	99.9819	99.98053	99.9727	99.96698	99.99852	99.93413
F1-Score	0.036189	0.038929	0.054595	0.066014	0.002956	0.131651
MCC	0.011464	0.011952	0.015908	0.017612	0.003779	0.025273
**Test case 3**						
Accuracy	90.77767	91.45067	92.668	93.945	96.61838	96.97933
Sensitivity	100	100	100	100	100	100
Specificity	90.77751	91.45052	92.66788	93.9449	96.61838	96.97928
Precision	0.018069	0.019491	0.022726	0.027518	0.001479	0.055145
FPR	9.222487	8.549476	7.332122	6.055101	3.381618	3.020717
FNR	0	0	0	0	0	0
NPV	90.77751	91.45052	92.66788	93.9449	96.61838	96.97928
FDR	99.98193	99.98051	99.97727	99.97248	99.99852	99.94485
F1-Score	0.036131	0.038974	0.045442	0.055021	0.002957	0.110229
MCC	0.012807	0.013351	0.014512	0.016078	0.00378	0.023126

In this study, the entire dataset is split into three equal test cases: test case 1, test case 2, and test case 3. Preprocessing is applied to each of these cases, resulting in preprocessing_1, preprocessing_2, and preprocessing_3. Similarly, the corresponding target labels are separated into target_1, target_2, and target_3. Finally, using fivefold cross-validation, each test case is validated. This approach ensures generalization and helps reduce overfitting. Here, the suggested model achieved as well as conventional approaches secured full sensitivity rate. Accuracy analysis performed on the developed intrusion detection model achieved 5.9% higher than SFO-AL-DBN, 6.42% better than JA-AL-DBN, 3.49% more effective than EHO-AL-DBN and 4.06% enhanced than AOA-AL-DBN in test case 1. Likewise, specificity analysis performed on the developed model achieved 6.8%, 6.04%, 4.6%, 3.2%, and 0.35% better than RF+SVM, DBN, LSTM, AL-DBN, and IVS-AVOA-HC, respectively. Here, proposed method achieves somewhat better accuracy compared to the IVS-AVOA-HC method due to parameter optimization in both the autoencoder and LSTM methods.

#### IVS-AVOA-HC

The algorithm is implemented based on the foraging and navigation behaviors of "African vultures" and consists of multiple phases like determining the best vulture, rate of vulture starvation, exploration, and exploitation. And, it is utilized to enhance the model’s efficacy by optimally selecting the optimal features and optimizing weight in the feature fusion phase.

#### MAOA-AL-HC

MAOA is implemented to optimize different parameters like epochs count in autoencoder and DBN, and hidden neuron count in LSTM and DBN for effective intrusion detection rate. The MAOA algorithm follows the AOA approach and involves initialization, volume, and density update, transfer operation, exploration, and exploitation phases.

Overall, both IVS-AVOA-HC and MAOA-AL-HC are adapted or developed to enhance the intrusion detection model’s performance. IVS-AVOA-HC focuses on feature selection and weight optimization, while MAOA-AL-HC addresses parameter optimization. The choice of which approach to use would depend on specific requirements, the complexities of the problem, and the desired balance between exploration and exploitation capabilities.

Thus, the proposed MAOA-AL-DBN-based intrusion detection model MAOA-AL-DBN secured a developed intrusion detection rate in IoT devices more than conventional models and also it gained an effective attack detection rate in IoT-based devices.

## 7. Conclusion

A new intrusion detection framework was developed based on deep learning structures to offer an efficient detection rate in IoT devices. Several data utilized for the analysis were acquired from the MQTT set and they are offered to pre-processing phase. Here, the input data were pre-processed by utilizing data cleaning and data transformation. Then, the pre-processed data were offered a feature extraction phase and acquired deep features from autoencoder and LSTM here two different features were attained. Later, two sets of the score were secured, the score 1 features were acquired from autoencoder, and also score 2 features were acquired from LSTM along with DBN, and their parameters were optimized with the help of developed MAOA. Then the acquired two different scores were offered to AL-DBN and averaging was performed to secure the intrusion detected outcome. Accuracy analysis performed on the developed intrusion detection model achieved 5.9% higher than SFO-AL-DBN, 6.42% better than JA-AL-DBN, 3.49% more effective than EHO-AL-DBN and 4.06% enhanced than AOA-AL-DBN in test case 1. Similarly, NPV analysis performed on the developed model achieved 5.4%, 4.2%, 2.9%, and 1.9% higher than SFO-AL-DBN, JA-AL-DBN, EHO-AL-DBN, and AOA-AL-DBN, respectively in 2^nd^ fold. Thus, the suggested intrusion detection model attained an effective intrusion detection rate than the existing model.

### Future scope

#### Further advancements in intrusion detection models

To enhance the proposed intrusion detection model, there is potential for exploring additional deep learning models and heuristic approaches. Researchers can investigate the effectiveness of advanced models such as convolutional neural networks (CNNs), recurrent neural networks (RNNs), or ensemble learning techniques. By incorporating these models, the performance of intrusion detection in IoT devices can be further improved.

#### Exploration of diverse datasets

While the research utilizes the MQTT set, exploring and incorporating other diverse and real-world IoT datasets would be beneficial. By working with different datasets, researchers can evaluate the generalizability and robustness of the proposed model across various IoT scenarios. This exploration will enable the identification of any limitations or challenges associated with different IoT data sources.

### MQTT set

In this study, a Kaggle public dataset containing records of assaults on the MQTT Protocol for IoT systems was utilized. The dataset used in the research can be accessed through the provided link.URL: https://www.kaggle.com/datasets/cnrieiit/mqttset
